# Impaired Odor Identification of Culturally Familiar Odorants Associated with Dementia in South Korean Older Adults

**DOI:** 10.3390/ijerph17228441

**Published:** 2020-11-14

**Authors:** Sun Mi Kim, Hye Ri Kim, Hyun Jin Min, Kyung Soo Kim, Hyuk Ga, Sang Hoon Lee, Doug Hyun Han

**Affiliations:** 1 Department of Psychiatry, Chung-Ang University College of Medicine, Seoul 06974, Korea; sunmikim706@caumc.or.kr (S.M.K.); et8536@gmail.com (H.R.K.); 2 Department of Otorhinolaryngology-Head and Neck Surgery, Chung-Ang University College of Medicine, Seoul 06974, Korea; jjinient@caumc.or.kr (H.J.M.); entkks@cau.ac.kr (K.S.K.); 3 Department of Family Medicine, Institute of Geriatric Medicine, Incheon Eun-Hye Hospital, Incheon 22711, Korea; gahyuk@gmail.com; 4 Department of Psychiatry, Eun-Hye Hospital, Incheon 22711, Korea; lnh409@naver.com

**Keywords:** neurocognitive disorders, odor identification, olfaction, clinical marker, Alzheimer’s disease

## Abstract

Among olfactory functions, odor identification is the most studied predictor of dementia. We aimed to verify whether patients with dementia are less aware of specific odors than cognitively normal individuals using an odor identification test, which includes odorants that are culturally familiar to South Koreans. We divided 139 older adults aged 57–79 years into the dementia and normal cognition groups. Odor identification function was assessed in all participants. We conducted hierarchical logistic regression analyses with the diagnosis of dementia as a dependent variable and three demographic characteristics, as well as 12 odor identification items, as independent variables. Impaired odor identification for herbal medicine (odds ratio (OR) = 9.420; *p* = 0.012) and Korean grilled meat (OR = 5.361; *p* = 0.019) and older age (OR = 1.176; *p* = 0.005) were significant predictors of dementia. Impaired odor identification of culturally familiar odorants was associated with dementia risk. This may be explained by the fact that compared with culturally non-specific universal odorants, familiar odorants are more related to episodic memory, which is impaired in the early stages of dementia. Thus, an optimal combination of odor identification items should be used for screening individuals with cognitive decline requiring further neurocognitive function tests.

## 1. Introduction

Among olfactory functions, odor identification is the most studied biomarker or predictor of Alzheimer’s disease (AD) [1,2,3,4]. Odor identification predicts dementia more accurately than odor threshold and discrimination, and it has the greatest correlation with cognitive function test results [5]. Since odor identification is impaired in the early stages of AD and odor threshold is damaged later, deficits in odor identification are prominent from the early stages of dementia, while a reduction in the odor threshold occurs after the disease progresses [6,7].

In a study in the United States, patients with AD were less aware of the smell of 5 out of the 40 items (i.e., bubble gum, root beer, watermelon, grass, and rose) in the University of Pennsylvania Smell Identification Test (UPSIT) [8] than healthy control subjects [9]. In a study conducted in the United States, 10 items predicted the conversion from mild cognitive impairment (MCI) to AD after a two-year follow-up, including menthol, clove, leather, strawberry, lilac, pineapple, smoke, soap, natural gas, and lemon [10]. Individuals who failed to identify the smell of these 10 items were at high risk for AD. In the United Kingdom, a study on AD-specific odors identified 12 items (motor oil, banana, coconut, apple, orange, cherry, watermelon, leather, cedar, pine, paint thinner, and peanut) that can be used to distinguish dementia from normal cognitive function with high sensitivity and specificity [11]. In a study conducted in Japan, the AD group showed impaired identification of odors such as India ink, wood, rose, Japanese cypress, and roasted garlic compared to the control group [12].

Since familiarity with odors differs on the basis of culture, race, and country, research on odors that are familiar to South Koreans is needed. In other words, studies need to be conducted to identify odors associated with dementia that include odors familiar to South Koreans. Therefore, this study aimed to verify whether patients with dementia are less aware of specific odors than the cognitively normal population using a standardized Korean olfactory function test, including odors culturally familiar to South Koreans. We hypothesized that specific odorants familiar to South Koreans would be associated with dementia in older South Koreans more effectively than culturally non-specific universal odorants.

## 2. Materials and Methods

### 2.1. Study Participants

From January 2019 to August 2019, participants were recruited at the Department of Psychiatry at Chung-Ang University Hospital, Seoul, South Korea. Through a hospital bulletin board, we recruited patients who were diagnosed with MCI or AD, those who visited the hospital to assess their cognitive decline, and individuals who were cognitively healthy. Participants aged < 80 years and their caregivers who understood the research process and provided written informed consent were included in the study. Exclusion criteria were (1) past or present neurological disorders or mental disorders except MCI and dementia; (2) past or present diagnosis of dementia except AD; (3) history of stroke or head trauma; (4) nasal-sinus illnesses or nasal-sinus operation; and (5) inability to communicate. The Institutional Review Board of Chung-Ang University Hospital approved this study. Written informed consent for inclusion and publication was obtained from all study participants and their caregivers before they participated in the study. The study was conducted in accordance with the Declaration of Helsinki, and the protocol was approved by the Institutional Review Board of Chung-Ang University Hospital (project identification code: 1811-001-309).

Since we planned to evaluate the predictive weight of 15 independent variables in two categories through logistic regression analyses with the diagnosis of dementia as a dependent variable, the total data of 119 participants in two groups (the normal cognition (NC) group and the dementia group) were needed according to an a priori sample size calculation (effect size (odds ratio) = 2, probability of error (significance level) α = 0.05, power of the test (1-β) = 0.90). Considering a 30% drop-out rate, 170 screenings were performed. Among the 170 individuals screened, 14 were excluded owing to poor understanding of the research protocol, inability to communicate, nasal-sinus illness, other neurologic or mental disorders, or a history of head trauma. The remaining 156 individuals were enrolled in the study. Finally, the data from 139 participants were statistically analyzed, excluding 17 participants with MCI.

### 2.2. Measurement

#### 2.2.1. Neurocognitive Test

The Korean Version of the Consortium to Establish a Registry for Alzheimer’s Disease Assessment Packet (CERAD-K) was conducted to assess the neurocognitive function of the participants [13]. The CERAD-K evaluates memory, attention, language, visuospatial function, and executive function. The CERAD-K scores ranged from 0 to 100. Diagnosis of MCI and major neurocognitive disorder (dementia) was made according to the DSM-5 [14]. Based on the clinical interview by psychiatrists and cognitive function assessed by the CERAD-K, participants were divided into the NC group (*n* = 99), the MCI group (*n* = 17), and the dementia group (*n* = 40). The NC and dementia groups were used for further analyses to identify specific odors to which patients with dementia showed impairment in the odor identification tests to predict dementia.

#### 2.2.2. YSK Olfactory Function Test

The YSK olfactory function test (RHICO Medical Co., Seoul, South Korea) was conducted to assess the olfactory functions of the participants [15]. This test assesses olfactory threshold, odor discrimination, and odor identification. In this study, only the odor identification test was performed. In the odor identification test, the participants were given 12 types of odorants, including universal odorants (baby powder, cinnamon, peaches, spearmint, chocolate, medicated patch, naphthalene, and ash) and odorants that are culturally familiar to South Koreans (herbal medicine, red ginseng, Korean grilled meat, and nurungji—the crust of overcooked rice). The odor stimuli were administered by felt-tip pens that were filled with liquid odorants and placed approximately 2 cm in front of both nostrils of the participant. The participants were asked to choose the identified odor from four alternatives. The correct answers were scored, and the total scores ranged from 0 to 12.

#### 2.2.3. Statistical Analysis

Differences in the sociodemographic characteristics, neurocognitive test scores, and the YSK odor identification test scores between the NC group and the dementia group were analyzed using the chi-square test and the independent *t*-test. To determine whether there was a significant association between neurocognitive function and odor identification function in this study population, we conducted preliminary hierarchical linear regression analyses with the CERAD-K score as the dependent variable. In the linear regression models, sex, age, and years of education were entered first (model 1), followed by the YSK odor identification test score (model 2). Next, we conducted hierarchical logistic regression analyses with the diagnosis of dementia as the dependent variable. In the logistic regression models, sex, age, and years of education were entered first (model 1), followed by 12 odor identification items (model 2). For all analyses, the level of significance was set at 0.05. All analyses were conducted using the Complex Samples module of the PASW statistics software package, version 19 (SPSS Inc., Chicago, IL, USA).

## 3. Results

### 3.1. Sociodemographic and Clinical Characteristics of the Study Population

Descriptive information on the sociodemographic factors and the levels of neurocognitive and odor identification functions are summarized in Table 1. The mean age of patients in the dementia group (74.15 ± 5.55 years) was significantly higher than that of patients in the NC group (68.48 ± 5.07 years; *t* = −5.801, *p* < 0.001). There were no differences in sex and level of education between the groups. The mean CERAD-K score in the dementia group (26.60 ± 13.58) was significantly lower than that in the NC group (74.18 ± 10.41; *t* = 19.920, *p* < 0.001). The mean YSK odor identification test score in the dementia group (5.28 ± 3.37) was significantly lower than that in the NC group (10.05 ± 2.66; *t* = 8.004, *p* < 0.001).

### 3.2. Results of the Hierarchical Linear Regression Analysis 

Results of the hierarchical linear regression analysis regarding the association of neurocognitive function with demographic factors and odor identification function are summarized in Table 2. Demographic factors, tested in model 1, explained 34.4% of the variance in neurocognitive function. Age (*p* < 0.001) and low level of education (*p* = 0.002) were negatively associated with the CERAD-K score. Odor identification function, tested in model 2, explained an additional 18.5% of the variance in neurocognitive function beyond the effects of demographic factors. The YSK odor identification score was positively associated with the CERAD-K score (*p* < 0.001). Additionally, in this final model, age (*p* < 0.001) and low level of education (*p* = 0.025) continued to independently show a negative relationship with the CERAD-K score. This final model explained 52.9% of the variance in neurocognitive function. The relative influence of the variables was estimated using the absolute value of the standardized coefficient β. The comparison showed that the variables influenced the CERAD-K score in the following order: YSK odor identification score (β = 0.514), age (β = −0.276), and level of education (β = −0.141).

### 3.3. Results of the Hierarchical Logistic Regression Analysis 

Models 1 and 2 of the hierarchical logistic regression analysis revealed a significant overall model fit (Table 3). In analysis model 1, the χ^2^ (33.462, *p* < 0.001) and Nagelkerke R^2^ value (0.306, explaining about 30.6% of variance in the dependent variable (dementia)) indicated that the model was acceptable for predicting dementia. When the practical usefulness of the model was examined based on the classification accuracy, 15 variables in analysis model 2 considerably enhanced its prediction accuracy up to 88.5% for the group membership of the dependent variable. Wald statistics were used to confirm whether each indicator had a significant individual relationship with dementia. Among the independent variables, older age (B = 0.162, Wald = 7.918, odds ratio = 1.176, *p* = 0.005) was a statistically significant predictor of dementia. In addition, impaired odor identification of herbal medicine (B = 2.243, Wald = 6.339, odds ratio = 9.420, *p* = 0.012) and Korean grilled meat (B = 1.679, Wald = 5.502, odds ratio = 5.361, *p* = 0.019) were statistically significant predictors of dementia.

## 4. Discussion

In summary, impaired odor identification of culturally familiar odorants, such as herbal medicine and Korean grilled meat, was associated with the risk of dementia even after adjusting for age. After accounting for the influence of other variables, impaired odor identification for herbal medicine increased the probability of dementia by 9.42-fold. Additionally, impaired odor identification for Korean grilled meat increased the probability of dementia by 5.36-fold.

In this study, odor identification function was positively associated with neurocognitive function beyond the effects of demographic factors, such as age and education level. These results are consistent with previous studies that reported a positive correlation between odor identification function and performance on neuropsychological tests [5]. Because odor identification requires a more complex cognitive process than odor threshold and discrimination, it has been suggested that the deficit in odor identification begins to appear in early AD, and gradually worsens as the disease progresses [5,16]. As a result of this preliminary analysis, we were able to confirm that our study participants were suitable to determine whether patients with dementia are less aware of specific odors than the cognitively normal population, including odors culturally familiar to South Koreans.

In this study, of the 12 odorants used to assess odor identification function, eight items were universal and four were familiar to South Koreans. Two odors familiar to South Koreans, such as herbal medicine and Korean grilled meat, were closely related to the risk of dementia. In Japan, a study comparing the applicability of two odor identification tests as biomarkers for AD showed that the test including all odorants familiar to the Japanese population showed a higher correlation with cognitive function scores than the other test containing some odorants not familiar to the Japanese population [17]. Identification of familiar odorants is dependent on both episodic memory and semantic memory, and identification of unfamiliar odorants is dependent only on semantic memory [18]. Episodic memories are conscious memories of personal experiences that comprise information about what happened and where and when it happened [19]. However, semantic memory is a part of long-term memory comprising general facts and conceptual knowledge, which are not acquired by personal experience [20]. The episodic memory function is very sensitive to brain aging, neurodegenerative diseases, and mental illnesses [21]. Impairment of episodic memory is an early symptom of AD; however, impairment of semantic memory occurs with the progression of dementia [22,23]. The brain networks for episodic memory and semantic memory share the same regions, such as the parahippocampal, middle temporal, ventroparietal, and medial prefrontal areas [23]. However, the hippocampus is only included in the episodic memory network [23]. Impairment of episodic memory in AD occurs because of the accumulation of neurite plaques and neurofibrillary tangles in the hippocampus and entorhinal cortex [24]. The entorhinal cortex is the hub of an extensive network for memory and perception, and receives fibers from the olfactory bulb [20]. Therefore, we may cautiously speculate that impaired odor identification for culturally familiar odorants is associated with AD because familiar odorants are more related to episodic memory, which is impaired from an earlier stage of dementia, than unfamiliar odorants or culturally non-specific odorants.

In this study, not all of the odorants that are culturally familiar to South Korean older adults had the same predictive weight, although impaired odor identification of herbal medicine and Korean grilled meat was statistically significantly associated with the risk of dementia, and that for red ginseng and crust of overcooked rice was statistically non-significant. Episodic memory is enhanced when experiences have affective valence, as the hippocampus interacts with the amygdala [25]. Therefore, for a particular odor to result in the construction of an episodic memory, the event may occasionally occur with autobiographical meaning and affective valence, not daily routine. Additionally, to show significant differences between groups when conducted on many study participants, the odor should not have been experienced by too few people. In Korea, the crust of overcooked rice may be a common odor frequently encountered in everyday life for anyone, and red ginseng may be a rare odor as an expensive medicinal ingredient. For this reason, it seems that the two items showed no statistical significance between the NC and dementia groups. In contrast, Korean grilled meat and herbal medicine are related to occasional events in Korea, for example, events of family dining out or going on picnics, or when a family member is sick, respectively. Therefore, these odors are thought to be more related to episodic memory and have a spastically significant predictive weight.

For the screening of dementia, conducting the full versions of existing odor identification tests is time-consuming. Considering the efficiency and cost in the clinical setting, an optimal combination of culturally familiar odorants, such as herbal medicine and Korean grilled meat that were related to dementia in this study, can be used to screen individuals with cognitive decline who require further neurocognitive function tests.

There are limitations in replicating the results of previous studies on odorants related to dementia, because the types, number, and odor intensity of odorants differ depending on the methods of the odor identification test. In previous studies, 40 items of the UPSIT were primarily used, whereas in this study, 12 items of the YSK olfactory function test were used for the odor identification test; hence, there may be other sensitive odorants for AD not included in the 12 items. Since this study was a single-center study for older adults in Seoul, South Korea, further multicenter studies with a large sample size in different cities of South Korea are necessary. Although people who have already been diagnosed with AD have been included and those with other types of dementia have been excluded, inclusion bias may have existed because neuroimaging or apolipoprotein E genotypes were not assessed for all participants. Although the analysis of this study excluded MCI patients because of the small number of MCI cases, the results of olfactory tests related to the risk of progressing from MCI to AD are also important. Therefore, longitudinal studies are needed to better understand the causal effect of the ability to identify specific odors on the risk of AD and on the risk of progression from MCI to AD. In addition, because of the associations between olfaction and anxiety and mood [26,27], it is necessary to measure the level of anxiety and depression among participants and to consider them as covariates in future studies.

## 5. Conclusions

Impaired odor identification for culturally familiar odorants, such as herbal medicine and Korean grilled meat, are associated with dementia in older South Korean individuals. The reason for this may be that familiar odorants are more related to episodic memory, which is impaired in the early stages of dementia, than culturally non-specific universal odorants.

## Figures and Tables

**Table 1:** Demographic characteristics of the study population (*n* = 139).

	Normal Cognition (*n* = 99)	Dementia (*n* = 40)	Statistics	*p-*Value
			*t*/χ^2^	
Age	68.48 ± 5.07	74.15 ± 5.55	*t* = −5.801	<0.001
Sex (male/female)	25/74 (25.3%/74.7%)	14/26 (35.0%/65.0%)	*t* = 1.341	0.247
Education			χ^2^ = 2.956	0.086
Below middle school	51 (51.5%)	27 (67.5%)		
High school graduate or higher	48 (48.5%)	13 (32.5%)		
CERAD-K score	74.18 ± 10.41	26.60 ± 13.58	*t* = 19.920	<0.001
YSK Odor identification score	10.05 ± 2.66	5.28 ± 3.37	*t* = 8.004	<0.001

Note. CERAD-K, Korean Version of the Consortium to Establish a Registry for Alzheimer’s Disease Assessment Packet.

**Table 2:** Results of linear regression analyses with the CERAD-K score as the dependent variable (*n* = 139).

Independent Variables		Model 1				Model 2			
		B	Beta	*t*	*p-*Value	B	Beta	*t*	*p-*Value
Demographic factors	Sex (female)	−1.071	−0.020	−0.274	0.785	−5.624	−0.104	−1.661	0.099
	Age, years	−2.164	−0.514	−7.180	<0.001	−1.164	−0.276	−3.996	<0.001
	Level of education (low)	−11.017	−0.225	−3.120	0.002	−6.921	−0.141	−2.264	0.025
Odor identification function	YSK Odor identification score					3.486	0.514	7.246	<0.001
Statistics of the model		F = 23.590 ***, R^2^ = 0.344				F = 37.567 ***, R^2^ = 0.529, F Change = 52.502 ***, R^2^ Change = 0.185			

Note. CERAD-K, Korean version of the Consortium to Establish a Registry for Alzheimer’s Disease Assessment Packet. *** *p* < 0.001.

**Table 3:** Results of hierarchical logistic regression analyses with diagnosis of dementia as a dependent variable for the normal cognition (*n* = 99) and dementia (*n* = 40) groups.

Independent Variables		Model 1				Model 2			
		B	Wald	*p-*Value	OR	B	Wald	*p-*Value	OR
Demographic factors	Sex (female)	−0.248	0.277	0.599	0.780	0.492	0.522	0.470	1.635
	Age, years	0.219	20.391	0.000	1.245	0.162	7.918	0.005	1.176
	Level of education (low)	0.557	1.559	0.212	1.746	−0.160	0.056	0.813	0.852
Odor identification (OI) (false for all items)	Item 1: baby powder					0.029	0.002	0.966	1.029
	Item 2: cinnamon					0.054	0.006	0.938	1.055
	Item 3: peaches					0.610	0.732	0.392	1.840
	Item 4: crust of overcooked rice					0.670	0.865	0.352	1.953
	Item 5: spearmint					0.770	1.210	0.271	2.160
	Item 6: chocolate					−1.623	3.175	0.075	0.197
	Item 7: herbal medicine					2.243	6.339	0.012	9.420
	Item 8: medicated patch					0.091	0.014	0.906	1.095
	Item 9: red ginseng					0.561	0.462	0.497	1.753
	Item 10: naphthalene					−1.446	3.580	0.058	0.236
	Item 11: Korean grilled meat					1.679	5.502	0.019	5.361
	Item 12: ash					1.171	2.225	0.136	3.226
Statistics of the model	−2LL		133.378				93.663		
	Step χ^2^		33.462 *** (df = 3)				39.717 *** (df = 12)		
	Model χ^2^		33.462 *** (df = 3)				73.179 *** (df = 15)		
	Nagelkerke R^2^		0.306				0.586		
	Classification accuracy		77.7%				88.5%		

Note. OR, odds ratio. *** *p* < 0.001.

## References

[1] Conti M.Z., Vicini-Chilovi B., Riva M., Zanetti M., Liberini P., Padovani A., Rozzini L. (2013). Odor identification deficit predicts clinical conversion from mild cognitive impairment to dementia due to Alzheimer’s disease. Arch. Clin. Neuropsychol..

[2] Devanand D.P. (2016). Olfactory identification deficits, cognitive decline, and dementia in older adults. Am. J. Geriatr. Psychiatry.

[3] Roberts R.O., Christianson T.J., Kremers W.K., Mielke M.M., Machulda M.M., Vassilaki M., Alhurani R.E., Geda Y.E., Knopman D.S., Petersen R.C. (2016). Association between olfactory dysfunction and amnestic mild cognitive impairment and Alzheimer disease dementia. JAMA Neurol..

[4] Sun G.H., Raji C.A., Maceachern M.P., Burke J.F. (2012). Olfactory identification testing as a predictor of the development of Alzheimer’s dementia: A systematic review. Laryngoscope.

[5] Djordjevic J., Jones-Gotman M., De Sousa K., Chertkow H. (2008). Olfaction in patients with mild cognitive impairment and Alzheimer’s disease. Neurobiol. Aging.

[6] Eibenstein A., Fioretti A.B., Simaskou M.N., Sucapane P., Mearelli S., Mina C., Amabile G., Fusetti M. (2005). Olfactory screening test in mild cognitive impairment. Neurol. Sci..

[7] Roalf D.R., Moberg M.J., Turetsky B.I., Brennan L., Kabadi S., Wolk D.A., Moberg P.J. (2017). A quantitative meta-analysis of olfactory dysfunction in mild cognitive impairment. J. Neurol. Neurosurg. Psychiatry.

[8] Doty R.L., Shaman P., Kimmelman C.P., Dann M.S. (1984). University of Pennsylvania Smell Identification Test: A rapid quantitative olfactory function test for the clinic. Laryngoscope.

[9] Warner M.D., Peabody C.A., Flattery J.J., Tinklenberg J.R. (1986). Olfactory deficits and Alzheimer’s disease. Biol. Psychiatry.

[10] Tabert M.H., Liu X., Doty R.L., Serby M., Zamora D., Pelton G.H., Marder K., Albers M.W., Stern Y., Devanand D.P. (2005). A 10-item smell identification scale related to risk for Alzheimer’s disease. Ann. Neurol..

[11] Velayudhan L., Gasper A., Pritchard M., Baillon S., Messer C., Proitsi P. (2015). Pattern of smell identification impairment in Alzheimer’s disease. J. Alzheimers Dis..

[12] Jimbo D., Inoue M., Taniguchi M., Urakami K. (2011). Specific feature of olfactory dysfunction with Alzheimer’s disease inspected by the Odor Stick Identification Test. Psychogeriatrics.

[13] Lee J.H., Lee K.U., Lee D.Y., Kim K.W., Jhoo J.H., Kim J.H., Lee K.H., Kim S.Y., Han S.H., Woo J.I. (2002). Development of the Korean version of the Consortium to Establish a Registry for Alzheimer’s Disease Assessment Packet (CERAD-K): Clinical and neuropsychological assessment batteries. J. Gerontol. B Psychol. Sci. Soc. Sci..

[14] American Psychiatric Association (2013). Diagnostic and Statistical Manual of Mental Disorders (DSM-5).

[15] Ha O.G., Kim J., Nam J.S., Park J.J., Cho H.J., Yoon J.H., Kim C.H. Normative values for the YSK olfactory function test and optimization for the diagnosis cut-off. Proceedings of the 25th Academic Conference of Otorhinolaryngology-Head and Neck Surgery.

[16] Kim H.R., Kim S.M., Seong W., Min H.J., Kim K.S., Ga H., Han D.H. (2020). Cut-off scores of an olfactory function test for mild cognitive impairment and dementia. Psychiatry Investig..

[17] Suzuki Y., Yamamoto S., Umegaki H., Onishi J., Mogi N., Fujishiro H., Iguchi A. (2004). Smell identification test as an indicator for cognitive impairment in Alzheimer’s disease. Int. J. Geriatr. Psychiatry.

[18] Oberg C., Larsson M., Bäckman L. (2002). Differential sex effects in olfactory functioning: The role of verbal processing. J. Int. Neuropsychol. Soc..

[19] Tulving E. (1983). Elements of Episodic Memory.

[20] Squire L.R., Dronkers N., Baldo J. (2009). Encyclopedia of Neuroscience.

[21] Kinugawa K., Schumm S., Pollina M., Depre M., Jungbluth C., Doulazmi M., Sebban C., Zlomuzica A., Pietrowsky R., Pause B.M. (2013). Aging-related episodic memory decline: Are emotions the key?. Front. Behav. Neurosci..

[22] Irish M., Lawlor B.A., O’Mara S.M., Coen R.F. (2011). Impaired capacity for autonoetic reliving during autobiographical event recall in mild Alzheimer’s disease. Cortex.

[23] Renoult L., Irish M., Moscovitch M., Rugg M.D. (2019). From knowing to remembering: The semantic-episodic distinction. Trends Cogn. Sci..

[24] Tchakoute C.T., Sainani K.L., Henderson V.W., Raloxifene in Alzheimer’s disease investigators (2017). Semantic memory in the clinical progression of Alzheimer’s disease. Cogn. Behav. Neurol..

[25] Sadock B.J., Sadock V.A., Ruiz P. (2017). Kaplan and Sadock’s Comprehensive Textbook of Psychiatry.

[26] Kamath V., Paksarian D., Cui L., Moberg P.J., Turetsky B.I., Merikangas K.R. (2018). Olfactory processing in bipolar disorder, major depression, and anxiety. Bipolar Disord..

[27] Siopi E., Denizet M., Gabellec M.M., de Chaumont F., Olivo-Marin J.C., Guilloux J.P., Lledo P.M., Lazarini F. (2016). Anxiety- and depression-like states lead to pronounced olfactory deficits and impaired adult neurogenesis in mice. J. Neurosci..

